# Structure, Morphology, and Photoelectric Performances of Te-Sb_2_Se_3_ Thin Film Prepared via Magnetron Sputtering

**DOI:** 10.3390/nano10071358

**Published:** 2020-07-11

**Authors:** Donglou Ren, Xue Luo, Shuo Chen, Zhuanghao Zheng, Michel Cathelinaud, Guangxing Liang, Hongli Ma, Xvsheng Qiao, Xianping Fan, Xianghua Zhang

**Affiliations:** 1 ISCR (Institut des Sciences Chimiques de Rennes)-CNRS, UMR 6226, Univ. Rennes, F-35000 Rennes, France; rendonglou15@mails.ucas.edu.cn (D.R.); xue.luo@univ-rennes1.fr (X.L.); michel.cathelinaud@univ-rennes1.fr (M.C.); hongli.ma@univ-rennes1.fr (H.M.); 2 State Key Laboratory of Silicon Materials & School of Materials Science and Engineering, Zhejiang University, Hangzhou 310027, China; qiaoxus@zju.edu.cn (X.Q.); fanxp@zju.edu.cn (X.F.); 3 Shenzhen Key Laboratory of Advanced Thin Films and Applications, College of Physics and Optoelectronic Engineering, Shenzhen University, Shenzhen 518060, China; chensh@szu.edu.cn (S.C.); zhengzh@szu.edu.cn (Z.Z.); lgx@szu.edu.cn (G.L.)

**Keywords:** Te-Sb_2_Se_3_ thin film, magnetron sputtering, structure, photoelectric performances

## Abstract

Antimony selenide (Sb_2_Se_3_) has been widely investigated as a promising absorber material for photovoltaic devices. However, low open-circuit voltage (V_oc_) limits the power conversion efficiency (PCE) of Sb_2_Se_3_-based cells, largely due to the low-charge carrier density. Herein, high-quality n-type (Tellurium) Te-doped Sb_2_Se_3_ thin films were successfully prepared using a homemade target via magnetron sputtering. The Te atoms were expected to be inserted in the spacing of (Sb_4_Se_6_)_n_ ribbons based on increased lattice parameters in this study. Moreover, the thin film was found to possess a narrow and direct band gap of approximately 1.27 eV, appropriate for harvesting the solar energy. It was found that the photoelectric performance is related to not only the quality of films but also the preferred growth orientation. The Te-Sb_2_Se_3_ film annealed at 325 °C showed a maximum photocurrent density of 1.91 mA/cm^2^ with a light intensity of 10.5 mW/cm^2^ at a bias of 1.4 V. The fast response and recovery speed confirms the great potential of these films as excellent photodetectors.

## 1. Introduction

The current most commercialized thin-film solar cells are copper indium gallium selenide (CIGS) and cadmium telluride (CdTe). Their market share is continuously reducing, mainly due to the scarcity of indium and gallium and the toxicity of cadmium [1,2]. In addition, the complex composition of CIGS is an issue for industrial production. To overcome these problems, many researchers have explored other earth-abundant and nontoxic absorber materials that consist of ribbons, for instance, antimony sulfide (Sb_2_S_3_) [3,4] and antimony selenide (Sb_2_Se_3_) [5,6,7]. The ribbons are held together by weak van der Waals forces. Once they are parallel to the grain boundary plane, the device performance can be significantly enhanced by eliminating dangling bonds at the grain boundary. Notably, the power conversion efficiency (PCE) of the Sb_2_Se_3_ solar cells has had a very rapid evolution within only 7 years, reaching 9.2% in 2019 based on the core–shell nanorod configuration [8]. This is ascribed to a series of excellent properties of this binary material, including a narrow band gap (1.1–1.3 eV), high absorption coefficient (>10^5^ cm^−1^) and fast carrier transport along the [001] orientation [5,8,9,10,11]. Moreover, the simple composition and the only stable phase in this binary system minimize the risk of impurity phase during thin film preparation. 

At present, the PCE of Sb_2_Se_3_ based solar cells is still limited by many poor characteristics such as its low-charge carrier density (~10^13^ cm^−3^) and high concentration of intrinsic point defects (vacancies and antisite defects) [10,12]. Thus far, some efficient ways to reduce the intrinsic point defects and prolong the minority carrier lifetimes in thin films are post-selenization [13], in-situ selenium compensation [14], and the use of vapor transport deposition [15]. It is well known that doping is an efficient approach to adjust the defects of semiconductors, which has been widely utilized in the semiconductor industry [16]. However, few studies have been carried out on doped Sb_2_Se_3_ thin films, and the development of appropriate doping for broadening the applications of this promising material is crucial. Since the notable PCE of 2.26% realized in 2014 [17], many methods of thin film preparation have been proposed [5,8,15,17,18,19]. Among these approaches, magnetron sputtering can realize high-quality thin film under a full-vacuum deposition condition [13,20,21], and the record PCE of 6.84% was reported by Liang et al in 2020 [22]. 

This work is on the preparation and characterization of Te-Sb_2_Se_3_ thin films prepared by using the sputtering method. We selected the element Te as a doping atom for different reasons. Firstly, our objective was to obtain a n-type semiconductor and Te has four valence electrons, one more than Sb. Secondly, it is known that Te can minimize defect formation in Sb_2_Se_3_ films [23], which may be correlated with its smaller atomic radius (1.43 Å) [24] in comparison with the gap of (Sb_4_Se_6_)_n_ ribbons (~3.5 Å) [25]. The structure, morphology, and photoelectric performances of these thin films were systematically investigated.

## 2. Materials and Methods 

High purity elements of antimony (Sb, 99.999%, Umicore), selenium (Se, 99.999%, Umicore), and Tellurium (Te, 99.999%, Umicore) were utilized as raw materials. 

### 2.1. Preparation of the Te-Sb_2_Se_3_ Target

The Te-Sb_2_Se_3_ target with the chemical composition of Sb_2_(Se_0.97_Te_0.03_)_3_ was prepared using a highly reproducible melting method at a high temperature. This composition has previously been optimized in a relationship in terms of its photoelectric properties. At first, a 70-g mixture was weighted in a glovebox under argon according to the chemical composition. Then, the mixture was loaded into a fully cleaned silica tube with an internal diameter of 50 mm. In order to eliminate all volatile substances in the mixture, the tube was evacuated and sealed until a vacuum of about 10^−5^ mbar was reached. Subsequently, the tube was placed in a rocking furnace and heated to 810 °C with a ramp rate of 1.2 °C/min. The mixture was continuously rocked at 810 °C for 10 h, after which the furnace was kept vertical for the crystallization of melt at a cooling rate of 1.2 °C/min. After cooling down to room temperature, the as-prepared specimen was carefully taken out of the silica tube and finely polished into a magnetron sputtering target with a thickness of around 6 mm and a diameter of 50 mm. 

### 2.2. Deposition of the Te-Sb_2_Se_3_ Thin Films

The Te-Sb_2_Se_3_ thin films were deposited by using a homemade target and a magnetron sputtering system (Plassys MP600S) equipped with an in-line optical monitoring system to measure the reflectance at a certain wavelength and the film thickness in real time. In addition, it can detect the crystallization process, which changes the reflection of the film. More detailed descriptions can be found in our previous works [26,27]. Indium tin oxide (ITO)-coated glasses (2.5 × 2.5 cm^2^) with a sheet resistance of 30 Ω were used as substrates and consecutively cleaned using detergent, acetone, ethanol, and deionized water for 15 min each. The sputtering chamber was evacuated to a pressure below 2 × 10^−7^ mbar before deposition. High-purity (99.99%) Ar gas with a mass flow rate of 30 sccm was used for depositing the thin films. The radio frequency (RF) sputtering power was kept at 12 W, and the working pressure was set at 0.04 mbar. Before deposition, the homemade target was pre-sputtered for 10 min to remove the contaminants from the surface. Afterwards, the thin films were deposited on unheated substrates that were continuously rotated during preparation. All thin films had a thickness of 350 nm, which was measured using the in-situ optical monitoring system and confirmed by observation under electronic microscope. Following that, the thin films were annealed in the range of 325–400 °C under a pressure of 0.1 mbar with 40 sccm flow of Ar for 90 min to induce crystallization. The temperature was monitored utilizing a thermocouple enclosed in the chamber.

### 2.3. Characterization

X-ray diffraction (XRD) with Cu Kα radiation was performed to analyze the crystalline structure on a PANalytical X-ray diffractometer in the range of 10–60° under 40 kV and 40 mA. The morphologies were observed by using a thermal field-emission scanning electron microscope (SEM, JEOL JSM-7100 F) equipped with an energy dispersive spectrometer (EDS). The optical properties of the thin films were characterized utilizing a PerkinElmer LAMBDA 1050 UV/Vis/NIR spectrophotometer with dual beam and a monochromator. The transmission and reflection measurements were performed at ambient temperature in a wavelength range of 500 to 1500 nm. The conductivity type of the thin films was determined via a Semilab PN tester PN-100. To evaluate the photoelectric performances of the thin films, photo-electro-chemical (PEC) measurements were carried out using a conventional three-electrode system in 0.5 M LiClO_4_ solution, where the thin films, a Pt-wire, and an Ag/AgCl electrode were used as the working, counter, and reference electrodes, respectively. A white light tungsten halogen lamp was used as a light source with a light intensity of 10.5 mW/cm^2^, and a detailed description can be found in our previous works [16,28].

## 3. Results and Discussion 

### 3.1. Target Characterization 

**Figure 1:**
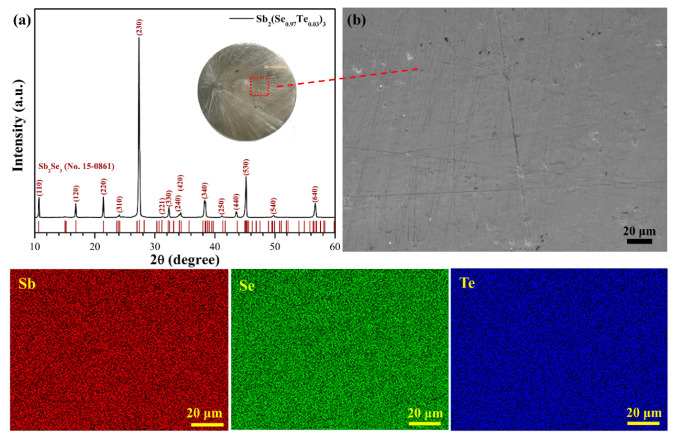
(**a**) The X-ray diffraction (XRD) pattern of the Te-Sb_2_Se_3_ target prepared by the melting method. The inset is the surface morphology. (**b**) Scanning electron microscope (SEM) image and energy dispersive spectroscopy (EDS) elemental mapping of Sb, Se, and Te.

Figure 1a shows the XRD pattern of the Te-Sb_2_Se_3_ target prepared by a melting method at a high temperature in vacuum. As observed, all diffraction peaks matched well with the orthorhombic phase of Sb_2_Se_3_ (JCPDS standard card 15-0861) without any noticeable impurities. The target exhibited a preferential crystallographic orientation of [230], largely due to the surface energy of some crystal planes during crystallization from liquid [29]. It was also observed that the polished target possessed a flat surface without cracks and porosity (Figure 1b), suggesting that the preparation method could completely meet the target demand of magnetron sputtering. In comparison with the commonly used spark plasma sintering method [30], the melting method could facilitate mass transport and thus improve the uniformity of the composition [31,32,33]. In addition, crystallization from a melt is generally exempt from porosity. Moreover, the distribution of elements was uniform, as evidenced by EDS elemental mapping, indicating that the Te element was well doped into the Sb_2_Se_3_ lattice. We prepared and characterized three targets and obtained the same results, confirming the repeatability of experiments. Meanwhile, the average composition of these targets is shown in Table 1. The atomic percentages of Sb, Se, and Te were 39.78 ± 0.28, 58.2 ± 0.17, and 2.02 ± 0.03at.%, respectively. 

### 3.2. Characterization on the Structure of Te-Sb_2_Se_3_ Thin Films 

**Figure 2:**
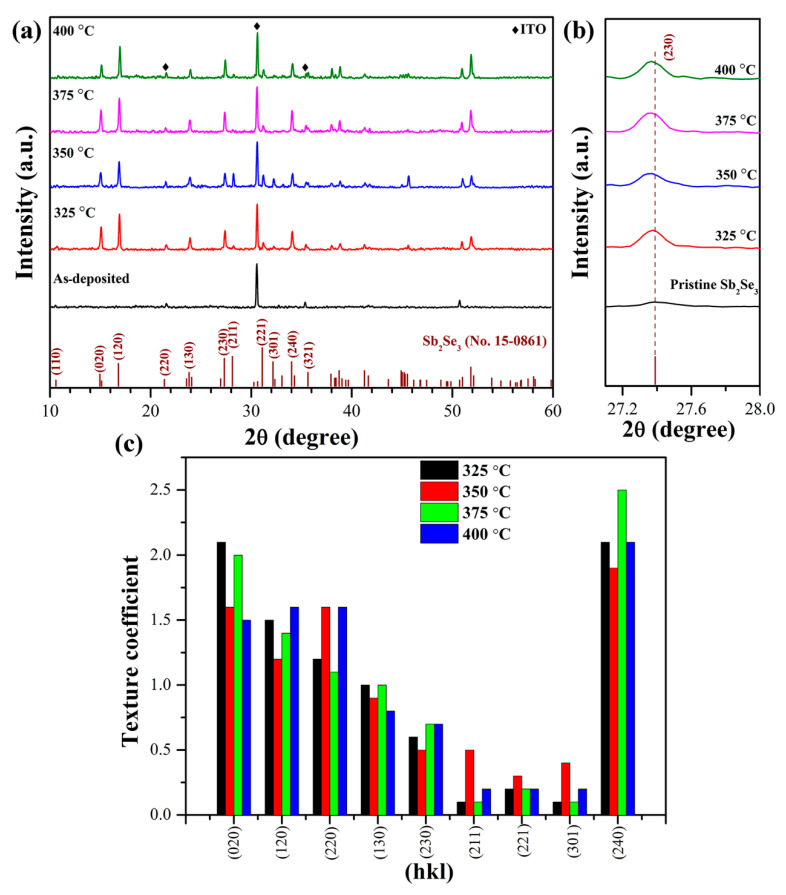
(**a**) XRD patterns of Te-Sb_2_Se_3_ thin films on indium tin oxide (ITO) with different annealing temperatures. (**b**) Shift of (230) peak of crystalline Te-Sb_2_Se_3_ thin films as shown in XRD patterns compared with pristine Sb_2_Se_3_. (**c**) Texture coefficients of diffraction peaks based on XRD patterns.

The XRD patterns of the as-deposited and annealed Te-Sb_2_Se_3_ thin films with different temperatures are presented in Figure 2a. For the as-deposited thin film, no crystalline diffraction peaks were detected, except for some peaks that originated from the ITO substrate, indicating the amorphous characteristic of the thin films deposited on an unheated substrate. Subsequently, the thin film was annealed in the range of 325–400 °C under Ar atmosphere with an interval of 25 °C. It is obvious that the emerging peaks are in good agreement with the orthorhombic Sb_2_Se_3_ (JCPDS standard card 15-0861). At the same time, the sharp diffraction peaks also indicate the highly crystalline nature of these annealed thin films. Most importantly, no impurity phases in any of the XRD patterns revealed that Te element was successfully doped into the lattice of Sb_2_Se_3_. To further confirm the successful doping behavior, we prepared pure Sb_2_Se_3_ thin film using the same deposition condition as a reference sample. As observed, the diffraction peak (230) slightly shifted toward a lower angle with Te doping (Figure 2b), which was due to an increase in the interplanar spacing according to Bragg’s equation. On the basis of a previous report [23] and by considering its electronic structure, Te cannot substitute for Se atoms in the Sb_2_Se_3_ lattice. Consequently, this observation implies that Te atoms are largely doped into the spacing of (Sb_4_Se_6_)_n_ ribbons (~3.5 Å) [25]. In addition, it is noteworthy that the intensity of (211) and (221) diffraction peaks first increased and then decreased, accompanied by the reverse trend for the (120) and (020) peaks. The thin film annealed at 350 °C had the highest intensity of (211) and (221) peaks as compared to other crystalline films. Generally, the crystallinity improved with an increasing annealing temperature. However, if the temperature was too high, e.g., 400 °C, the intensity of diffraction peaks showed a slight decrease, which is attributed to the deterioration of the films due to quite high vapor pressure of Sb_2_Se_3_ at this temperature [5]. 

It is well known that film orientation is crucial for Sb_2_Se_3_ thin film solar cells. To quantify the difference of preferential orientations between different Te-Sb_2_Se_3_ thin films at different annealing temperatures, the texture coefficient (TC) of diffraction peaks was calculated using the following equation:

(1) $$TC_{\mathrm{hkl}}=\frac{I_{\left(\mathrm{hkl}\right)}}{I_{0(\mathrm{hkl})}}/(\frac{1}{N}\sum_{N}\frac{I_{\left(\mathrm{hkl}\right)}}{I_{0(\mathrm{hkl})}}),$$

where *I*_(hkl)_ is the measured diffraction peak intensity of (hkl) plane and *I*_0(hkl)_ is the intensity value in the standard XRD pattern. *N* is the total number of planes considered for the calculation. In general, a larger TC value implies a preferred orientation of grain in films [18]. As observed in Figure 2c, the film annealed at 350 °C exhibited the strongest overall TC values for the (hk1) planes, further indicating the desired orientation for photovoltaic applications.

**Figure 3:**
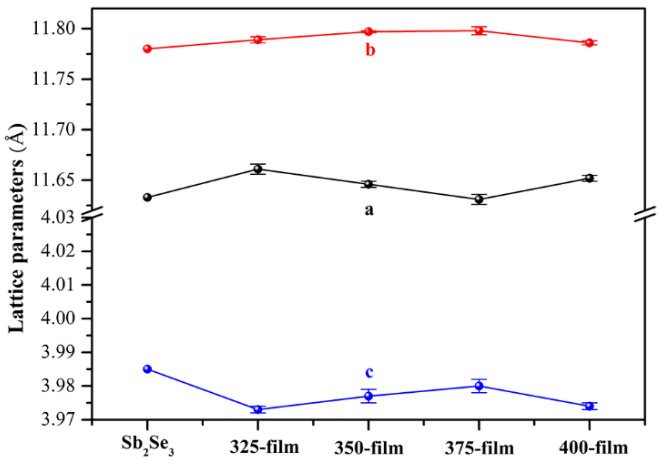
The lattice parameters of Te-Sb_2_Se_3_ thin films at different annealing temperatures and pure Sb_2_Se_3_.

To better understand the mechanism of Te doping, the lattice parameters of Te-Sb_2_Se_3_ thin films were calculated using JADE 6.0 software, as shown in Figure 3. Referring to the different annealing temperatures, the thin films were denoted as 325-film, 350-film, 375-film, and 400-film, respectively. Obviously, the lattice parameter *a* (or *b*) of Te-Sb_2_Se_3_ thin films slightly increased, while the lattice parameter c slightly decreased when compared with pure Sb_2_Se_3_ [34]. It suggests that the lattice parameter along the directions that the (Sb_4_Se_6_)_n_ ribbons were held together through weak van der Waals forces [6], i.e., the [100] and [010] axes increased remarkably. For an orthorhombic crystal, it is well known that the relationship between interplanar spacing (*d*_hkl_) and lattice constants (*a*, *b*, *c*) can be expressed as follows [34]:

(2) $$d_{\mathrm{hkl}}=\frac{1}{{\left[{\left(\frac{h}{a}\right)}^{2}+{\left(\frac{k}{b}\right)}^{2}+{\left(\frac{1}{c}\right)}^{2}\right]}^{0.5}},$$

As a result, increased lattice parameters resulted in broadened interplanar spacing and the (230) diffraction peak shifted toward a lower angle (Figure 2b). Moreover, compared with the intrinsic p-type nature of pure Sb_2_Se_3_ thin film, the conductivity type of Te-Sb_2_Se_3_ thin films was n-type, as verified by the PN tester. Considering the larger lattice parameters and the n-type doping behavior, we thus further believe that Te element was mainly located in the gap between the (Sb_4_Se_6_)_n_ ribbons and was a donor in the Sb_2_Se_3_ film. Such a phenomenon has been frequently found for Cd and Na doping in Sb_2_Se_3_ as well [6,25].

**Figure 4:**
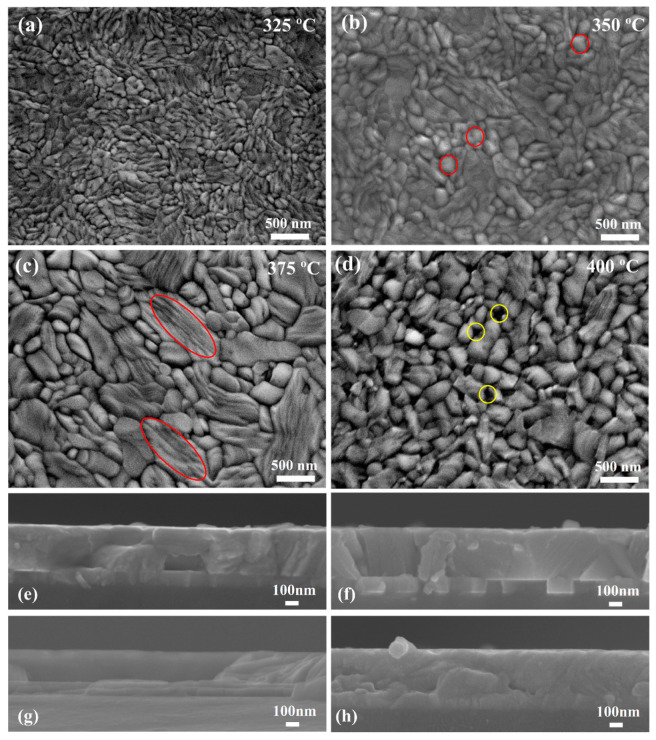
Top-views and cross-section SEM images of the Te-Sb_2_Se_3_ thin films annealed at temperatures of (**a**,**e**) 325, (**b**,**f**) 350, (**c**,**g**) 375, and (**d**,**h**) 400 °C.

Figure 4 displays scanning electron microscopy (SEM) images of the Te-Sb_2_Se_3_ thin films with different annealing temperatures. For 325 °C-annealed thin film, small crystal grains were apparent (Figure 4a). In contrast, the grain size increased in the 350 °C-annealed thin film, accompanied by many round grains (red circles, Figure 4b), and thus the intensities of diffraction peaks (211) and (221) were higher (Figure 2a). Notably, the film annealed at 350 °C was compact and free of cracks. When the annealing temperature increased to 375 °C, the morphology of film was considerably different from the 350 °C-annealed thin film; a large number of rod grains (red ellipses, Figure 4c) appeared along with large melted/softened areas. The intensity of diffraction peak (120) was enhanced; however, the (211) and (221) orientations were reduced (Figure 2a). Meanwhile, the 375 °C crystalline film showed loose grain boundaries due to thermal etching, as already observed in similar materials [26]. By further increasing the temperature to 400 °C, the film significantly deteriorated, leading to the formation of apparent holes (yellow circles, Figure 4d) and sparse surfaces due to excessive thermal etching. Therefore, the annealing temperature plays a crucial role in achieving high-quality Te-Sb_2_Se_3_ film. As seen in Figure 4e-h, a good interface quality without cracks was generally observed.

### 3.3. Optical Properties of Te-Sb_2_Se_3_ Thin Films

**Figure 5:**
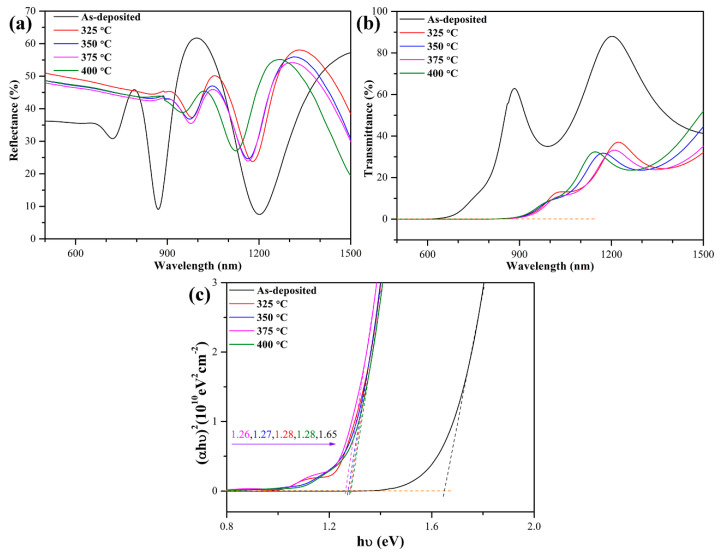
Optical characterizations of Te-Sb_2_Se_3_ thin films at different annealing temperatures. (**a**) Reflection spectra, (**b**) Transmission spectra, and (**c**) Plot of (*αhυ*)^2^ vs. *hυ* for obtaining the direct band gap.

In order to further investigate the optical properties of the Te-Sb_2_Se_3_ thin films, an UV/Vis/NIR spectrophotometer was used to measure the reflection and transmission at room temperature in a wavelength range of 500–1500 nm with glass as the substrate. As observed in Figure 5a, the reflectance of the as-deposited thin film was much lower than that of the crystalline thin films in the short wavelength region, mainly due to its density-dependent lower refractive index as well as its disorder. Moreover, the red-shift of the short wavelength cut-off edge from 640 nm for the as-deposited thin film to around 876 nm for the crystalline thin film was evident, resulting in the evolution of the band gap. To further evaluate the effect of Te doping on the band structure, the band gap width (*E*_g_) of the thin films was calculated using the following formulas:

(3) $$\alpha =\frac{1}{d}\ln\left(\frac{1-R\left(\lambda \right)}{T\left(\lambda \right)}\right),$$

(4) $$\alpha hv=B{\left({hv-E}_{g}\right)}^{r},$$

where *α* is the absorption coefficient; *d* is thickness of thin film (350 nm); and *R* and *T* represent the reflectance and transmittance, respectively [35]. Equation (4) is a classical Tauc relationship, where *B* is a constant; *υ* is the photo frequency; *h* is the Planck’s constant; and *r* = 2 for an indirect band gap semiconductor and *r* = 0.5 for a direct band gap semiconductor [4]. As shown in Figure 5c, the as-deposited thin film possessed a direct band gap of 1.65 eV; the value remarkably decreased to around 1.27 eV after annealing due to the change in atomic arrangement from disorder to order. A slight increase in the band gap width compared with pure Sb_2_Se_3_ thin film (1.15 eV) [36] was observed after Te doping, which is ascribed to the combination of the Burstein–Moss shift and the renormalization effect, existing in the narrow band gap semiconductor with a certain doping level [37,38]. On the whole, the band gap of the Te-Sb_2_Se_3_ thin films is still suitable for efficiently harvesting visible light.

### 3.4. Photoelectric Performances of Te-Sb_2_Se_3_ Thin Films

**Figure 6:**
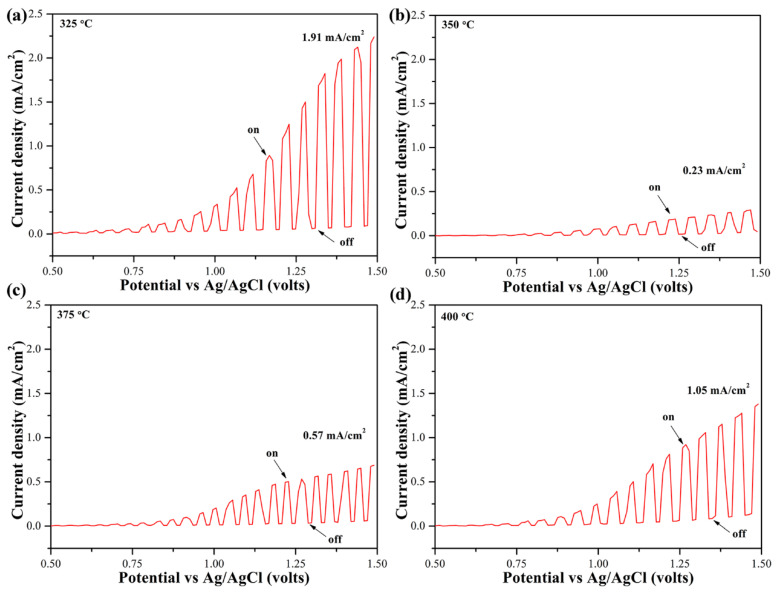
Photocurrent-potential response curves of Te-Sb_2_Se_3_ films with different annealing temperatures: (**a**) 325, (**b**) 350, (**c**) 375, and (**d**) 400 °C.

To evaluate the PEC performance of the Te-Sb_2_Se_3_ films annealed at different temperatures, photo-electro-chemical measurements were performed under chopped light with an intensity of 10.5 mW/cm^2^. As can be observed in Figure 6, all Te-Sb_2_Se_3_ films showed anodic photocurrents, and their photocurrent density increased with the positive bias, indicating that Te-Sb_2_Se_3_ films are n-type semiconductors, which is in agreement with the result obtained from the PN tester. There was no apparent variation in the photocurrent response at the potential between 0.5 and 0.75 V. It is well known that Sb_2_Se_3_ apparently shows direction-dependent bonding characteristics. The carrier transport in the [001] direction occurred much easily than in the [221] direction, in which the (Sb_4_Se_6_)_n_ ribbons were tilted at a smaller angle with the substrate [8]. Compared with the 350 °C-annealed Te-Sb_2_Se_3_ film, the reduced intensity of the diffraction peaks (221) and (211) for other crystalline films (Figure 2a) implies that they grew with improved preference along the [001] direction (c-axis), resulting in higher photocurrent density. In addition, the photocurrent density obviously depends on the quality of the films. As a result, a maximum photocurrent density of 1.91 mA/cm^2^ at around 1.4 V was obtained for the film annealed at 325 °C. On the contrary, the lowest response of 0.23 mA/cm^2^ was observable for 350 °C-annealed film. Moreover, Te was shown to significantly reduce deep level defects, due to the improved conductivity and reduced recombination, leading to a longer carrier lifetime [23]. As a result, the Te-Sb_2_Se_3_ film was shown to possess much better photoelectric properties than pure Sb_2_Se_3_ film.

**Figure 7:**
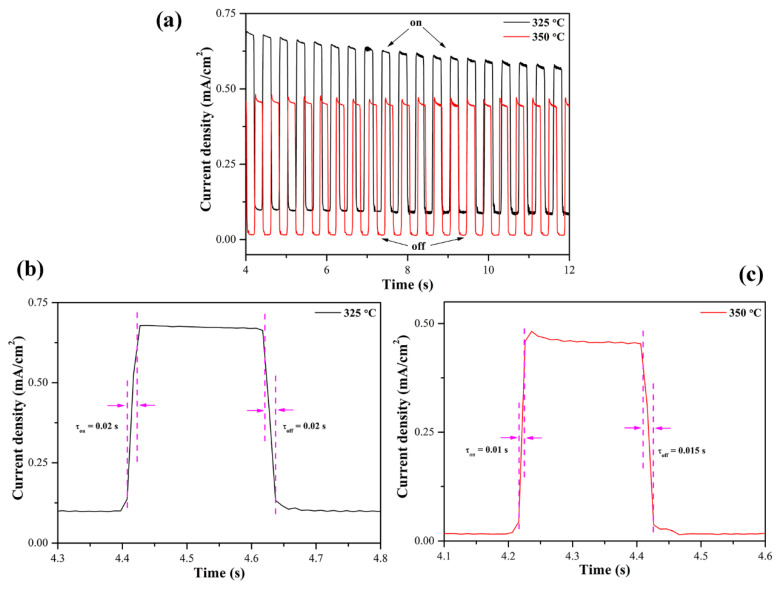
(**a**) Time-resolved PEC photoresponse of Te-Sb_2_Se_3_ films annealed at 325 and 350 °C. (**b**) and (**c**) show the response and recovery times, respectively.

The cyclic photoresponse of the Te-Sb_2_Se_3_ films was also characterized, as shown in Figure 7a. Clearly, the photocurrent density of the Te-Sb_2_Se_3_ film annealed at 325 °C showed a slight decline during several cycles, while the 350 °C-annealed Te-Sb_2_Se_3_ film was stabilized almost at its original level. This is attributed to the low-density morphology that makes the 325 °C film more susceptible to oxydo-reduction. The response (τ_on_) and recovery (τ_off_) times are two important parameters that can be used to assess the speed of the on/off switching, which is defined as the time between 10% and 90% values of the maximum current. As observed in Figure 7b and c, the 350 °C film shows a shorter response time of 0.01 s and recovery time of 0.015 s, which is ascribed to higher crystal quality, preferential orientation, and the grain size [16]. Furthermore, the discrepancy in response/recovery times may result from the traps and other defects [39], leading to increased recombination of photogenerated carriers in the 350 °C film. Notably, the response time of the Te-Sb_2_Se_3_ film was shorter than that mentioned in many previous literatures [39,40], suggesting its high application potential as excellent photodetectors.

## 4. Conclusions

N-type Sb_2_Se_3_ was obtained through Te doping, and high-quality thin films were prepared by using magnetron sputtering. It was found that post-deposition annealing has a crucial influence on the properties of the films. Different investigations showed that Te atoms are inserted into the spacing of (Sb_4_Se_6_)_n_ ribbons as donors based on increased lattice parameters. All of the films maintained a narrow band gap of approximately 1.27 eV, ideal for harvesting the solar energy. The photoelectric performance of the films was also found to be highly dependent on the annealing temperatures, and the film annealed at 325 °C realized a maximum photocurrent density of 1.91 mA/cm^2^ with a light intensity of 10.5 mW/cm^2^ at a bias of 1.4 V. The fast response and strong photocurrent density give these films the potential to be used as photodetectors as well as for photovoltaic application.

## Figures and Tables

**Table 1:** The energy dispersive spectroscopy (EDS) results of the Te-Sb_2_Se_3_ targets prepared by the melting method in a vacuum (average composition).

Element	Sb	Se	Te
Atomic percent (at.%)	39.78 ± 0.28	58.2 ± 0.17	2.02 ± 0.03

## References

[1] (2020). Photovoltaics Report. https://www.ise.fraunhofer.de/content/dam/ise/de/documents/publications/studies/Photovoltaics-Report.pdf.

[2] Lei H., Chen J., Tan Z., Fang G. (2019). Review of recent progress in antimony chalcogenide-based solar cells: Materials and devices. Sol. RRL.

[3] Zhang L., Jiang C., Wu C., Ju H., Jiang G., Liu W., Zhu C., Chen T. (2018). V_2_O_5_ as hole transporting material for efficient all inorganic Sb_2_S_3_ solar cells. ACS Appl. Mater. Interfaces.

[4] Luo J., Xiong W., Liang G., Liu Y., Yang H., Zheng Z., Zhang X., Fan P., Chen S. (2020). Fabrication of Sb_2_S_3_ thin films by magnetron sputtering and post-sulfurization/selenization for substrate structured solar cells. J. Alloys Compd..

[5] Liu X., Chen J., Luo M., Leng M., Xia Z., Zhou Y., Qin S., Xue D.J., Lv L., Huang H. (2014). Thermal evaporation and characterization of Sb_2_Se_3_ thin film for substrate Sb_2_Se_3_/CdS solar cells. ACS Appl. Mater. Interfaces.

[6] Zhou Y., Li Y., Luo J., Li D., Liu X., Chen C., Song H., Ma J., Xue D.-J., Yang B. (2017). Buried homojunction in CdS/Sb_2_Se_3_ thin film photovoltaics generated by interfacial diffusion. Appl. Phys. Lett..

[7] Ma C., Guo H., Wang X., Chen Z., Cang Q., Jia X., Li Y., Yuan N., Ding J. (2019). Fabrication of Sb_2_Se_3_ thin film solar cells by co-sputtering of Sb_2_Se_3_ and Se targets. Sol. Energy.

[8] Li Z., Liang X., Li G., Liu H., Zhang H., Guo J., Chen J., Shen K., San X., Yu W. (2019). 9.2%-efficient core-shell structured antimony selenide nanorod array solar cells. Nat. Commun..

[9] Tang R., Chen X., Luo Y., Chen Z., Liu Y., Li Y., Su Z., Zhang X., Fan P., Liang G. (2020). Controlled sputtering pressure on high-quality Sb_2_Se_3_ thin film for substrate configurated solar cells. Nanomaterials.

[10] Chen C., Bobela D.C., Yang Y., Lu S., Zeng K., Ge C., Yang B., Gao L., Zhao Y., Beard M.C. (2017). Characterization of basic physical properties of Sb_2_Se_3_ and its relevance for photovoltaics. Front. Optoelectron..

[11] Ren D., Merdrignac-Conanec O., Dorcet V., Cathelinaud M., Zheng Z., Ma H., Zhang X. (2020). In situ synthesis and improved photoelectric performances of a Sb_2_Se_3_/β-In_2_Se_3_ heterojunction composite with potential photocatalytic activity for methyl orange degradation. Ceram. Int..

[12] Huang M., Xu P., Han D., Tang J., Chen S. (2019). Complicated and unconventional defect properties of the quasi-one-dimensional photovoltaic semiconductor Sb_2_Se_3_. ACS Appl. Mater. Interfaces.

[13] Tang R., Zheng Z.H., Su Z.H., Li X.J., Wei Y.D., Zhang X.H., Fu Y.Q., Luo J.T., Fan P., Liang G.X. (2019). Highly efficient and stable planar heterojunction solar cell based on sputtered and post-selenized Sb_2_Se_3_ thin film. Nano Energy.

[14] Liu X., Xiao X., Yang Y., Xue D.-J., Li D.-B., Chen C., Lu S., Gao L., He Y., Beard M.C. (2017). Enhanced Sb_2_Se_3_ solar cell performance through theory-guided defect control. Prog. Photovolt. Res. Appl..

[15] Tao J., Hu X., Guo Y., Hong J., Li K., Jiang J., Chen S., Jing C., Yue F., Yang P. (2019). Solution-processed SnO_2_ interfacial layer for highly efficient Sb_2_Se_3_ thin film solar cells. Nano Energy.

[16] Chen S., Qiao X., Zheng Z., Cathelinaud M., Ma H., Fan X., Zhang X. (2018). Enhanced electrical conductivity and photoconductive properties of Sn-doped Sb_2_Se_3_ crystals. J. Mater. Chem. C.

[17] Zhou Y., Leng M., Xia Z., Zhong J., Song H., Liu X., Yang B., Zhang J., Chen J., Zhou K. (2014). Solution-processed antimony selenide heterojunction solar cells. Adv. Energy Mater..

[18] Li K., Chen C., Lu S., Wang C., Wnag S., Lu Y., Tang J. (2019). Orientation engineering in low-dimensional crystal-structural materials via seed screening. Adv. Mater..

[19] Yuan C., Zhang L., Liu W., Zhu C. (2016). Rapid thermal process to fabricate Sb_2_Se_3_ thin film for solar cell application. Sol. Energy.

[20] Liang G.-X., Zheng Z.-H., Fan P., Luo J.-T., Hu J.-G., Zhang X.-H., Ma H.-L., Fan B., Luo Z.-K., Zhang D.-P. (2018). Thermally induced structural evolution and performance of Sb_2_Se_3_ films and nanorods prepared by an easy sputtering method. Sol. Energy Mater. Sol. Cells.

[21] Luo Y.-D., Tang R., Chen S., Hu J.-G., Liu Y.-K., Li Y.-F., Liu X.-S., Zheng Z.-H., Su Z.-H., Ma X.-F. (2020). An effective combination reaction involved with sputtered and selenized Sb precursors for efficient Sb_2_Se_3_ thin film solar cells. Chem. Eng. J..

[22] Liang G.-X., Luo Y.-D., Chen S., Tang R., Zheng Z.-H., Li X.-J., Liu X.-S., Liu Y.-K., Li Y.-F., Chen X.-Y. (2020). Sputtered and selenized Sb_2_Se_3_ thin-film solar cells with open-circuit voltage exceeding 500 mV. Nano Energy.

[23] Ma Y., Tang B., Lian W., Wu C., Wang X., Ju H., Zhu C., Fan F., Chen T. (2020). Efficient Defect Passivation of Sb_2_Se_3_ Film by Tellurium Doping for High Performance Solar Cells. J. Mater. Chem. A.

[24] Said S.M., Bashir M.B.A., Sabri M.F.M., Miyazaki Y., Shnawah D.A.A., Hakeem A.S., Shimada M., Bakare A.I., Ghazali N.N.N., Elsheikh M.H. (2017). Enhancement of thermoelectric behavior of La_0.5_Co_4_Sb_12−x_Te_x_ skutterudite materials. Metall. Mater. Trans. A.

[25] Li Y., Zhou Y., Luo J., Chen W., Yang B., Wen X., Lu S., Chen C., Zeng K., Song H. (2016). The effect of sodium on antimony selenide thin film solar cells. RSC Adv..

[26] Chen S., Zheng Z., Cathelinaud M., Ma H., Qiao X., Su Z., Fan P., Liang G., Fan X., Zhang X. (2019). Magnetron sputtered Sb_2_Se_3_-based thin films towards high performance quasi-homojunction thin film solar cells. Sol. Energy Mater. Sol. Cells.

[27] Ren D., Chen S., Cathelinaud M., Liang G.-X., Ma H., Zhang X. (2020). Fundamental physical characterization of Sb_2_Se_3_-based quasi-homojunction thin film solar cells. ACS Appl. Mater. Interfaces.

[28] Ren D., Zheng Z., Wei M., Zhang P., Cathelinaud M., Ma H., Zhang X. (2020). Synthesis, structure and photoelectric properties of selenide composites with in situ constructed Sb_2_Se_3_/NaSbSe_2_ heterojunction. J. Eur. Ceram. Soc..

[29] Zhou Y., Wang L., Chen S., Qin S., Liu X., Chen J., Xue D.J., Luo M., Cao Y., Cheng Y. (2015). Thin-film Sb_2_Se_3_ photovoltaics with oriented one-dimensional ribbons and benign grain boundaries. Nat. Photonics.

[30] Liang G., Chen X., Tang R., Liu Y., Li Y., Luo P., Su Z., Zhang X., Fan P., Chen S. (2020). Spark plasma sintering of Sb_2_Se_3_ sputtering target towards highly efficient thin film solar cells. Sol. Energy Mater. Sol. Cells.

[31] Ren D., Deng Q., Wang J., Li Y., Li M., Ran S., Du S., Huang Q. (2017). Densification and mechanical properties of pulsed electric current sintered B_4_C with in situ synthesized Al_3_BC obtained by the molten-salt method. J. Eur. Ceram. Soc..

[32] Ren D., Deng Q., Wang J., Yang J., Li Y., Shao J., Li M., Zhou J., Ran S., Du S. (2018). Synthesis and properties of conductive B_4_C ceramic composites with TiB_2_ grain network. J. Am. Ceram. Soc..

[33] Wang J., Ren D., Chen L., Man G., Zhang H., Zhang H., Luo L., Li W., Pan Y., Gao P. (2020). Initial investigation of B_4_C–TiB_2_ composites as neutron absorption material for nuclear reactors. J. Nucl. Mater..

[34] Tian Y., Sun Z., Zhao Y., Zhang Y., Tan T., Yin F. (2019). Facile spray drying approach to synthesize Sb_2_Se_3_/rGO composite anode for lithium-ion battery. J. Nanoparticle Res..

[35] Kobayashi T., Kumazawa T., Kao Z.J.L., Nakada T. (2013). Cu(In,Ga)Se_2_ thin film solar cells with a combined ALD-Zn(O,S) buffer and MOCVD-ZnO:B window layers. Sol. Energy Mater. Sol. Cells.

[36] Chen C., Li W., Zhou Y., Chen C., Luo M., Liu X., Zeng K., Yang B., Zhang C., Han J. (2015). Optical properties of amorphous and polycrystalline Sb_2_Se_3_ thin films prepared by thermal evaporation. Appl. Phys. Lett..

[37] Walsh A., Da Silva J.L.F., Wei S.H. (2008). Origins of band-gap renormalization in degenerately doped semiconductors. Phys. Rev. B Condens. Matter Mater. Phys..

[38] Gibbs Z.M., LaLonde A., Snyder G.J. (2013). Optical band gap and the Burstein-Moss effect in iodine doped PbTe using diffuse reflectance infrared Fourier transform spectroscopy. New J. Phys..

[39] Liu Y.-Q., Zhang M., Wang F.-X., Pan G.-B. (2014). Facile microwave-assisted synthesis of uniform Sb_2_Se_3_ nanowires for high performance photodetectors. J. Mater. Chem. C.

[40] Zhai T., Ye M., Li L., Fang X., Liao M., Li Y., Koide Y., Bando Y., Golberg D. (2010). Single-crystalline Sb_2_Se_3_ nanowires for high-performance field emitters and photodetectors. Adv. Mater..

